# The clinical burden of cow's milk allergy in early childhood: A retrospective cohort study

**DOI:** 10.1002/iid3.572

**Published:** 2021-12-06

**Authors:** Katy Sorensen, Rosan Meyer, Kate E. Grimshaw, Abbie L. Cawood, Dionisio Acosta‐Mena, Rebecca J. Stratton

**Affiliations:** ^1^ Medical Affairs, Nutricia Ltd. Trowbridge UK; ^2^ Department of Paediatrics St. Mary's Hospital London UK; ^3^ Dietetic Department, Salford Care Organisation Salford Royal NHS Foundation Trust Salford UK; ^4^ Institute of Human Nutrition, Faculty of Medicine, University of Southampton Southampton General Hospital Southampton UK; ^5^ Cegedim Health Data, Cegedim Rx London UK

**Keywords:** cow's milk allergy, functional gastrointestinal disorders, infants, infections, pediatrics, primary care

## Abstract

**Introduction:**

Cow's milk allergy (CMA) is common in infants and children. Clinical presentations may vary, with a range of symptoms affecting the gastrointestinal (GI), skin and respiratory systems. Whilst the primary focus of research to date has been on the management of these symptoms, studies investigating the broader clinical burden of CMA are limited.

**Methods:**

We performed a retrospective matched cohort study examining clinical data, including allergic symptoms and infections, extracted from case records within The Health Improvement Network database. A total of 6998 children (54% male) were included in the study, including 3499 with CMA (mean age at diagnosis 4.04 months) and 3499 matched controls without CMA, observed for a mean period of 4.2 years.

**Results:**

GI, skin and respiratory symptoms affected significantly more children with CMA (*p* < .001), which recurred more often (*p* < .001), compared with children without CMA. More children with CMA had symptoms affecting multiple systems (*p* < .001). CMA was associated with a greater probability of these symptoms requiring hypoallergenic formula (HAF) prescription persisting over time (log‐rank test *p *< .0001, unadjusted hazard ratio [HR]: 0.81, 95% confidence interval [CI]: 0.76–0.85, *p* < .001), with a longer median duration of symptoms and HAF prescription compared with the duration of symptoms in those without CMA (3.48 vs. 2.96 years). GI, skin, respiratory and ear infections affected significantly more children with CMA than those without, increasing by 74% (*p *< .001), 20% (*p* < .001), 9% (*p* < .001), and 30% (*p* < .001) respectively. These infections also recurred more often among children with CMA, increasing by 62% for GI infections, 37% for skin and respiratory infections, and 44% for ear infections (*p* < .001).

**Conclusions:**

This real‐world study provides evidence to suggest that CMA presents a significant clinical burden to children, which has implications for the healthcare system. Further research is warranted to understand the health economic impact of this, and the phenotypes, factors and management approaches which may affect clinical outcomes.

## Graphical abstract

This large, real‐world, restrospective cohort study provides novel evidence of a significant clinical burden of CMA in children. Children with cow's milk allergy (CMA) have significantly more gastrointestinal, skin and respiratory symptoms, lasting significantly longer, than children without CMA. Additionally, children with CMA have significantly more gastrointestinal, skin, respiratory and ear infections.

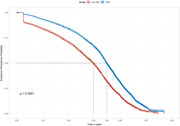

## 1 INTRODUCTION

Cow's milk allergy (CMA) is a common food allergy during childhood, typically presenting within the first year of life among 2‐5% of infants in Europe.^1^, ^2^, ^3^, ^4^ It involves a hypersensitive immune‐mediated reaction to cow's milk protein (CMP).^1^ The avoidance of CMP is therefore the hallmark of CMA management, while ensuring nutritional adequacy.^5^ This should ideally include breastmilk, and where not available, hypoallergenic formula (HAF), including extensively hydrolyzed formula (eHF) which are usually suggested as first‐line feeds with amino‐acid formula (AAF) being used for severe CMA or when symptoms remain unresolved with eHF.^1^, ^3^, ^6^, ^7^

CMA can be IgE, non‐IgE, or mixed IgE and non‐IgE mediated.^1^, ^3^ Approximately 44% of CMA cases are thought to be IgE mediated,^2^ which is associated with an immediate onset of symptoms typically affecting one or more organ systems within minutes to an hour of exposure.^1^ Conversely, up to 56% of CMA cases may be non‐IgE mediated,^2^ which may be even greater in the United Kingdom.^8^ These cases tend to present with delayed gastrointestinal (GI) with or without skin or respiratory symptoms, which can appear several days after CMP exposure.^1^

A combination of upper and lower GI symptoms are usually observed such as reflux, vomiting, soft stool constipation, diarrhea, perianal dermatitis, presence of blood or mucus in stools, abdominal distension, colic and in some cases faltering growth. Skin symptoms such as erythema, urticaria, angio‐edema and eczema are common and respiratory symptoms may also be present, including allergic rhinitis, cough, wheeze, asthma, and anaphylaxis in rare and severe cases.^9^

To date, the primary research focus has been on the resolution of the typical CMA associated symptoms, but not on the broader burden of this condition. Increased susceptibility to infections, such as upper respiratory tract^10^ and ear infections,^11^ has been documented in some observational studies of children with food allergies, along with lower IgA and deviated IgG classes, which may be suggestive of immunodeficiency.^10^, ^12^ This may have a significant impact on infants, families, and the healthcare system. This real‐world retrospective cohort study aimed to compare the clinical burden, including symptoms and infections, of children with CMA to those without.

## 2 METHODS AND MATERIALS

### 2.1 Study design

This retrospective cohort study compared case records from The Health Improvement Network (THIN, A Cegedim Proprietary Database) of children with CMA compared with children without CMA. Similar methodologies using the THIN database have been cited in more than 1000 research publications to date.^13^

At the time the study was conducted, the THIN database contained anonymized longitudinal records of 2.9 million active patient records from 365 general practices in the United Kingdom. Demographic and clinical information is documented within the THIN database using read‐codes. These have been used by healthcare professionals since 1985, and provide a coded thesaurus of clinical terms.^14^ Information relating to prescriptions is documented using the World Health Organisation index of Anatomical Therapeutic Chemical (ATC) codes.^15^ Predefined codes can be extracted from the THIN database to provide full patient histories including their demographics, clinical symptoms, procedures, prescriptions, diagnoses, healthcare professional referrals and contacts, providing a generalizable insight into real‐world clinical practice in the United Kingdom.^16^

### 2.2 Study population

Data were extracted on the 4th November 2020 from 6998 anonymised case records indexed within the last five years. This included 3499 children with confirmed or suspected CMA at ≤12 months of age. Confirmed CMA was defined by a CMA diagnosis read‐code. Suspected CMA, in the absence of a CMA diagnosis read‐code, was defined by the prescription of a HAF for at least three consecutive months. A cohort of 3499 children without CMA (matched for age, sex, and index of multiple deprivation [IMD]) were also included. Exclusion criteria aimed to omit children receiving HAF for documented conditions outside of allergy, and those with conditions which could impact on clinical outcomes. This included:

- Children with read‐codes for intestinal failure; necrotizing enterocolitis; cancer, malignancy or tumor; congenital heart disease; cystic fibrosis; cerebral palsy; metabolic conditions; chromosomal anomalies.
- Children prescribed any other medical nutrition product not indicated for CMA.

### 2.3 Study variables and outcome measures

Demographic data was extracted from case records including age, sex, country of residence, IMD (quintiles 1 [least deprived] to 5 [most deprived] calculated from the IMD score distribution),^17^, ^18^, ^19^, ^20^ ethnicity, presence of other allergies, and family history of allergies. Data on breastfeeding was not reliably recorded within the data set and therefore could not be included. Clinical outcome data included symptoms, selected from the National Institute for Health and Care Excellence (NICE) Clinical Knowledge Summary for CMA,^9^ and infections. Symptom data included overall GI symptoms (reflux, vomiting, diarrhea, constipation, flatulence, blood in stools, mucus in stools, colic, general GI illness, and faltering growth), overall skin symptoms (eczema, urticaria, and erythema), overall respiratory symptoms (asthma and rhinitis of any type) and anaphylaxis. Infection data included GI, skin, respiratory, and ear infections. GI infections included viral gastroenteritis, gastroenteritis of other presumed infectious origin, campylobacter GI infection, and diarrhea and vomiting caused by suspected infection. Skin infections included skin and subcutaneous tissue infections. Respiratory infections included upper respiratory tract infection and acute tonsilitis. Ear infections included otitis media, infective otitis externa, and ear pain.

### 2.4 Statistical analysis

Outcomes were measured from birth over the duration of available data for each child (referred to as the observation period throughout) (mean: 4.2 years [range: 3.5–5.8] for both cohorts). Results were presented primarily as the number (*n*) and proportion (%) of children who had the outcome at least once during the observation period. Outcome data were also presented as rates per 5‐person‐years, to estimate the average number of times that each child in the cohort would experience the outcome during a five‐year period. Rates per 5‐person‐years were calculated by dividing the total number of events for a specific outcome by the total number of years over which the children were observed during the study, then multiplying by five.

Statistical analysis was performed using R software, version 4.0.2.^21^ Statistical significance was set at *p* < .05. Between group differences in proportional data were measured using the Fisher's exact or *χ* ^2^ test of independence, as appropriate. Between group differences in rates were measured using the Poisson test. A Kaplan–Meier model was used to estimate the probability for the outcome of achieving at least three months of no symptoms (and no HAF prescription). Probability (survival) curves were generated from the model to compare the probability distribution of symptom persistence requiring HAF among the CMA group, and symptom persistence among the non‐CMA group. Their differences were compared using the log‐rank test. Median duration of symptoms (and HAF prescription for the CMA group) was also estimated for each group. A Cox proportional hazard regression model was used to determine crude (unadjusted) hazard ratios (HR) for the persistence of symptoms and HAF among CMA group, compared with the persistence of symptoms among the comparator group of children without CMA.

## 3 RESULTS

### 3.1 Characteristics

Cohort characteristics are presented in Table 1. Groups were matched for age, sex and IMD. There were some statistically significant demographic differences between the CMA and non‐CMA groups. This included country of residence, with a higher proportion children in Northern Ireland having CMA than not, and ethnicity, where the majority of the CMA group were white (of note, overall the majority of case records did not contain data on ethnicity, Table 1). As expected, more children with CMA had records of “other” allergies and a family history of allergy, compared with those without.

**Table 1:** Cohort characteristics

Characteristic	CMA (*n* = 3499)	non‐CMA (*n* = 3499)	*p*‐value
Male, *n* (%)	1896 (54)	1896 (54)	>.9
Country of residence, *n* (%)			<.001
England	968 (28)	1285 (37)	
Northern Ireland	607 (17)	385 (11)	
Scotland	978 (28)	1033 (30)	
Wales	946 (27)	796 (23)	
IMD quintile, *n* (%)			.071
5th	776 (23)	788 (23)	
4th	916 (27)	915 (27)	
3rd	597 (18)	546 (16)	
2nd	378 (11)	449 (13)	
1st	743 (22)	726 (21)	
Ethnicity, *n* (%)			<.001
White	1207 (93)	1265 (87)	
Mixed/multiple ethnic groups	17 (1.3)	31 (2.1)	
Asian/Asian British	51 (3.9)	85 (5.8)	
Black/Black British	19 (1.5)	59 (4.0)	
Other	10 (0.8)	17 (1.2)	
Presence of “other” allergy^a^, *n* (%)	547 (16)	184 (5.3)	<.001
Family history of allergy^b^, *n* (%)	55 (1.6)	25 (0.7)	.001

Abbreviations: CMA, cow's milk allergy; IMD, index of multiple deprivation, 5th, most deprived; 1st, least deprived.

^a^ Including read‐codes documented in case records for egg allergy, peanut allergy, food allergy, history of drug allergy, history of nondrug allergy and allergic reaction unspecified.

^b^ Including read‐codes documented in case records for family history of allergic disorders, allergy, atopy, eczema and hay fever.

A read‐code for CMA was present for 29% of the CMA group, with the remainder assigned to this group due to having a HAF prescription for at least three consecutive months. Of the CMA group, all were prescribed HAF (mean: 122 [SD: 35.6] g/day, for a mean of 9.5 [SD: 9.1] months) of whom 88% were prescribed eHF and 35% AAF, indicating that some children had both feeds prescribed during the observation period. The mean age of CMA diagnosis (defined as age at recording of a CMA read‐code or first HAF prescription) was 4.04 (SD: 2.79) months.

### 3.2 Symptoms

#### 3.2.1 GI, skin and respiratory symptoms

During the observation period, GI, skin and respiratory symptoms occurred in both groups, but affected significantly more children in the CMA cohort than those in the non‐CMA cohort (Table 2). These differences were statistically significant for all types of symptoms, except for blood in stools (*p* = .073), mucus in stools (*p*= .6) and anaphylaxis (*p* = .12), which were infrequently recorded in both cohorts. Significantly more infants with CMA had symptoms which affected multiple organ systems, compared with those without CMA (28% vs. 14%, *p* < .001).

**Table 2:** Differences in the proportion of children with GI, skin, respiratory, and multisystem symptoms in the CMA versus non‐CMA cohort

	CMA (*n* = 3499)	Non‐CMA (*n* = 3499)	*p*‐value
Overall GI symptoms, *n* (%)	2262 (65)	1463 (42)	<.001
Reflux	534 (15)	116 (3.3)	<.001
Vomiting	907 (26)	519 (15)	<.001
Diarrhea	977 (28)	609 (17)	<0001
Constipation	843 (24)	545 (16)	<.001
Flatulence	29 (0.8)	9 (0.3)	.002
Blood in stools	29 (0.8)	16 (0.5)	.073
Mucus in stools	3 (<0.1)	1 (<0.1)	.6
Colic	317 (9.1)	95 (2.7)	<.001
General GI illness	530 (15)	238 (6.8)	<.001
Faltering growth	75 (2.1)	27 (0.8)	<.001
Overall skin symptoms, *n* (%)	1286 (37)	841 (24)	<.001
Eczema	1215 (35)	776 (22)	<.001
Urticaria and erythema	140 (4)	92 (2.6)	.002
Overall respiratory symptoms, *n* (%)	274 (7.8)	140 (4.0)	<.001
Asthma	250 (7.1)	132 (3.8)	<.001
Rhinitis	25 (0.7)	12 (0.3)	.048
Anaphylaxis	6 (0.2)	1 (<0.1)	.12
Proportion of infants with multiple systems affected (GI/respiratory/skin), *n* (%)			
<2 systems affected	2522 (72%)	3011 (86%)	<.001
≥2 systems affected	977 (28%)	488 (14%)	

Abbreviations: CMA, cow's milk allergy; GI, gastrointestinal.

Additionally, the rate of overall GI, skin and respiratory symptoms per 5‐person‐years was significantly higher among children with CMA compared with those without CMA (Table 3). GI symptoms were the most common in both cohorts, and children with CMA had twice as many episodes of GI, skin and respiratory symptoms than those without.

**Table 3:** Differences in the rate of GI, skin, respiratory and anaphylaxis symptoms in the CMA versus non‐CMA cohort

Symptom rate per 5 person‐years	CMA (*n* = 3499)	Non‐CMA (*n *= 3499)	*p*‐value
Overall GI symptoms	2.15	1.00	<.001
Reflux	0.27	0.06	<.001
Vomiting	0.48	0.24	<.001
Diarrhea	0.50	0.28	<.001
Constipation	0.49	0.29	<.001
Flatulence	0.01	0.005	.004
Blood in stools	0.01	0.005	.041
Mucus in stools	<0.000	<0.000	.625
Colic	0.13	0.04	<.001
General GI illness	0.23	0.10	<.001
Overall skin symptoms	1.05	0.55	<.001
Eczema	0.98	0.51	<.001
Urticaria and erythema	0.06	0.04	<.001
Overall respiratory symptoms	0.20	0.10	<.001
Asthma	0.16	0.08	<.001
Rhinitis	0.01	0.005	.023
Anaphylaxis	<0.000	<0.000	.070

Abbreviations: CMA, cow's milk allergy; GI, gastrointestinal.

**Figure 1:**
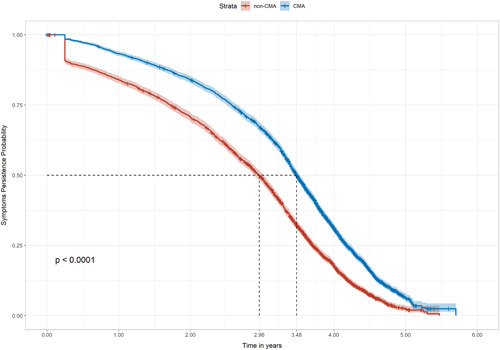
Kaplan–Meier analysis of the probability of symptom persistence over the observation period (log rank: *p* < .0001). CMA, cow's milk allergy

Kaplan–Meier analysis showed that the CMA cohort had a significantly greater probability of these symptoms requiring HAF prescription persisting over time (log‐rank test, *p* < .0001, unadjusted HR: 0.81, 95% confidence intervals: 0.76–0.85, *p* < .001). For children with CMA, this was associated with a longer median duration of symptoms and HAF prescription, compared with the median duration of symptoms among those without CMA (3.48 vs. 2.96 years for the non‐CMA cohort, Figure 1).

#### 3.2.2 Infections

During the observation period, GI, skin, respiratory and ear infections occurred in both groups. Respiratory infections were the most common, followed by skin infections. Table 4 illustrates that all categories of infections significantly affected more children with CMA and at a greater frequency per 5 person‐years, compared with children without CMA (*p* < .001). Overall, 92% (*n* = 3208) of children with CMA suffered from an infection compared with 86% (*n *= 3001) in the non‐CMA cohort (*p* < .001).

**Table 4:** Differences in occurrence of infections in the CMA versus non‐CMA cohort

	CMA (*n* = 3499)	Non‐CMA (*n *= 3499)	*p*‐value
GI infections			
*n* (%)^a^	282 (8.1)	162 (4.6)	<.001
Infection rate^b^	0.105	0.065	<.001
Skin infections			
*n* (%)^a^	1898 (54)	1584 (45)	<.001
Infection rate^b^	1.305	0.955	<.001
Respiratory infections			
*n* (%)^a^	3098 (89)	2854 (82)	<.001
Infection rate^b^	6.88	5.03	<.001
Ear infections			
*n* (%)^a^	875 (25)	673 (19)	<.001
Infection rate^b^	0.51	0.355	<.001

Abbreviations: CMA, cow's milk allergy; GI, gastrointestinal.

^a^ Proportion of children with at least one infection during observation period.

^b^ Per 5‐person‐years.

## 4 DISCUSSION

To our knowledge, this is the first large real‐world cohort study to compare the clinical burden of children with CMA to those without. This data, from nearly 7000 children who were observed for more than 4 years on average, shows that children with CMA not only suffer from more symptoms, but also face a significantly greater infectious burden than children without CMA, providing valuable insights into the clinical experiences of children with CMA in the United Kingdom.

Interesting differences in the demographic distribution of cohorts were present. A higher proportion of the CMA cohort resided in Northern Ireland, compared with the non‐CMA cohort. This may be a reflection of local training programs in recent years, leading to increased awareness of CMA and changes to prescribing practice among GPs. “Other” allergies and family history of allergy were also more prevalent among the CMA cohort, which is consistent with previous studies which have identified these as risk factors for CMA.^22^, ^23^, ^24^

As expected, GI, skin and respiratory symptoms were present in both cohorts, with such symptoms commonly experienced during infancy as part of immune development.^25^ Recent research suggests that 5‐20% of infants have colic,^26^ with several possible aetiologies hypothesized in addition to CMA.^27^, ^28^ Similarly, reflux and functional constipation affect 30%–67% and 3%–27% of infants respectively and may be behavioral, physiological or atopic in etiology.^26^ Skin rashes are also common in infants, accounting for 20%–30% of primary care visits.^29^ Whilst a proportion of eczema cases may be non‐atopic,^30^ there is a greater prevalence of CMA among children with infantile eczema.^31^ Similarly, atopic dermatitis may present in 11%–20% of children in the United Kingdom,^32^ and has been found to be more common in children with CMA than those without.^33^ The presentation of these symptoms in children with and without CMA may lead to challenges with differential diagnosis in clinical practice. This invites future research about the prognostic impact of the timing of CMA diagnosis and management.

In the present study, whilst symptoms were common to both cohorts, those with CMA had significantly more symptoms compared with children without CMA. GI symptoms affected 55% more children with CMA, occurring 115% more often. The greatest increases in prevalence were seen in reflux and colic. Skin symptoms affected more children with CMA, with increases of 57% in eczema and 52% in urticaria and erythema, recurring 91% more often overall. Respiratory symptoms affected 96% more children with CMA, increasing by 108% for rhinitis and 89% for asthma, recurring 100% more frequently overall. Moreover, the proportion of children with symptoms affecting multiple organ systems was also significantly higher among the CMA cohort, indicating a greater cumulative burden of symptoms, compared with those without.

Importantly, these symptoms persisted over a significantly longer period in children with CMA, compared with those without, equating to an additional 6.2 months (+18%) median duration of symptoms. This persistence of allergic symptoms in those with CMA, beyond what might be expected and observed in those without CMA, supports previous findings in the literature. Indeed, the concept of an “allergic march” has been described extensively, relating in particular to the role of atopic dermatitis as a trigger for the later development of respiratory conditions such as asthma and rhinitis^34^, ^35^, ^36^, ^37^ and of gastrointestinal conditions including irritable bowel syndrome.^38^ Studies suggest that 50%–75% of children with CMA will outgrow their allergy by 2 years of age, although this may vary with the type of CMA.^39^, ^40^, ^41^, ^42^ Whilst it is not possible to determine CMA outgrowth from the THIN database, the findings of this study may support the hypothesis of an early role of CMA in the allergic march,^35^ with an ongoing clinical impact beyond its outgrowth.

These findings have implications for allergic children, their families and the healthcare system. Functional gastrointestinal symptoms (FGIDs) such as colic, constipation and regurgitation may have a personal burden and lead to economic costs for families and healthcare systems.^26^ Furthermore, allergic symptoms are correlated with lower quality of life^43^ and psychosocial burden,^44^ which may be exacerbated by delayed diagnosis. Indeed, there have been reports of dissonance between GP and parental experiences of allergic conditions within the literature, with parents of children with eczema feeling that the psychosocial impact of symptoms is overlooked by GPs,^45^ and over half of parents of children with CMA stating that they were made to feel like they were over‐reacting or “worrying too much about nothing.”^44^ These findings support the notion that the clinical experiences of children with CMA are significantly different from those of children without CMA, and may therefore help to reconcile discrepant perspectives between parent and physician of the clinical burden of CMA.

A novel and important finding of this study was that a range of infections occurred in significantly more children with CMA, more often, compared with children without CMA. The greatest increases were found in GI infections, occurring in 74% more children, followed by ear, skin and respiratory infections (30%, 20%, and 9% increases, respectively). Respiratory infections are the most common infection in early childhood,^46^ which may explain the smaller differences between groups. However, children with CMA also had more frequent episodes of infection, with 62% more GI infections, 44% more ear infections and 37% more skin and respiratory infections respectively.

Several studies have recognized the association between atopic conditions and infection, finding an increased susceptibility to infections of bacterial, fungal and viral origin in children.^47^ Ear infections have previously been reported in children with allergic conditions^11^, ^48^, ^49^ and, among those with food protein‐induced gastrointestinal allergy, 68% have been reported to have frequent upper respiratory tract infections occurring more than once a month and lasting longer than their siblings.^10^

The dualistic mechanisms between allergic and infectious illnesses are not completely understood.^50^ Ear infections are a common manifestation of reflux in young infants,^51^ which was more common in the CMA cohort and may suggest a non‐atopic mechanism. There is also speculation of an inhibitive interaction between allergic inflammation and antiviral cytokines, which may account for the concurrence of chronic otitis media in allergic rhinitis^52^ and viral infections in atopic asthma.^50^ Indicators of immunodeficiencies including deviated levels of serum immunoglobulin‐A, immunoglobulin‐G subclasses and lymphocytes among children with allergy^10^, ^12^ may suggest an inadequate immune response. This may be further affected by gut bacteria^12^ which influences the development and maintenance of immune homeostasis, and defends against pathogenic colonization.^53^, ^54^, ^55^ Gut dysbiosis is prevalent among infants with CMA^56^, ^57^, ^58^, ^59^, ^60^ and may contribute to their increased incidence of infections.^53^ Studies have shown reductions in infections associated with the modification of the dysbiotic gut microbiota, with the use of an AAF containing pre‐ and probiotics (synbiotics).^57^, ^61^ This invites further research into this potential therapeutic target for immune health in allergic disease.

This study has a number of limitations. Whilst the prevalence and frequency of symptoms may vary in the first 3 years of life,^8^ changes in specific symptoms over time were not investigated, causing difficulty in distinguishing symptoms associated with allergy from those of common FGIDs which may be outgrown in early life. There was also no differentiation between symptoms before or following CMA diagnosis, with no investigation of the impact of the timing of diagnosis on clinical outcomes, which warrants further research. Furthermore, read‐codes may only be recorded when the patient presents to the clinician, such as for a repeat prescriptions, leading to potential underreporting of recurrent episodes.

Indeed, variations in recording practices may have contributed to differences between the data generated in this study, and that reported elsewhere. “Other” allergies were recorded in a small proportion of the CMA cohort, whereas they have been observed in 91% of patients with IgE‐mediated CMA.^24^ Similarly, whilst there is up to 72% chance of atopy in infants when present in both parents, family history of allergy was infrequently recorded, suggesting this information is not routinely documented in primary care. Emergency attendance for anaphylaxis may not be transferred to GP records, which may explain why incidence was low and similar between groups. Blood and mucus in stools are common in children with CMA,^62^ but were infrequently recorded in the present study, possibly due to limited read‐codes. Whilst asthma is a recognized comorbidity of CMA^9^ and present in 13.5% of the general population of children in England at four years of age,^63^ overall prevalence was low in the present study. This may reflect challenges with diagnosis in young children,^63^ warranting the inclusion of alternative read‐codes, such as cough or wheeze, to increase sensitivity of data analysis. However, this would reduce the specificity of the results, as such symptoms could have allergic or infectious causes. Therefore, where possible, read‐codes for comorbidities (such as asthma and eczema) were used instead of generic symptoms (such as wheeze and rash) to differentiate between manifestations of CMA and infection, which itself presents further challenges in clinical practice. However, a similar margin of error may apply across the cohort, affecting absolute values but not necessarily differences between groups.

Absence of read‐codes prevented the analysis of other symptoms of interest (such as angioedema, perianal dermatitis and specific classifications of rhinitis) and IgE status, which may be correlated with the timing of CMA outgrowth.^39^ CMA outgrowth itself was also not recorded, leading to uncertainty as to whether persistent symptoms related to ongoing CMA or manifestations of the allergic march. CMA diagnosis read‐codes were also infrequently recorded throughout the THIN database. HAF prescription for at least three months was therefore used as a diagnostic proxy for the CMA cohort, in the absence of a read‐code. Chronic conditions were excluded as described in the methodology, which is expected to have omitted children requiring HAF for chronic conditions outside of allergy^64^ and for a 2–4 week diagnostic elimination diets.^9^ However, this pragmatic approach meant that all of the CMA cohort had a HAF prescription. As breastfeeding is recommended first‐line in allergic infants, this may not reflect actual clinical practice, and could highlight potentially confounding differences between groups. However, HAF prescription may have been exclusive or additive to breastfeeding, and exclusive breastfeeding may have been practiced before or after HAF prescription. Breastfeeding is not commonly coded in GP databases, and published statistics on breastfeeding in CMA are lacking. In the general UK population, by four months of age (comparable with the mean age of CMA diagnosis and/or first HAF prescription in the CMA cohort) 12% of infants are exclusively breastfed,^65^ suggesting many children in the non‐CMA group may have been formula‐fed. Therefore, whilst different distributions of ethnic groups^66^, ^67^ and country of residence^65^ may lead to some variations between groups, similar rates of exclusive breastfeeding may be assumed across the cohorts. Further research to better understand breastfeeding practices in CMA is warranted.

## 5 CONCLUSIONS

Overall, the findings of this study are suggestive of a heightened clinical burden among children with CMA that may have additional implications for the healthcare system. Previous research has demonstrated an extensive impact of allergic conditions on UK healthcare services, with substantial associated costs. This large real‐world cohort study provides novel evidence of a significant clinical burden of CMA in children. Additional research is required to further quantify the healthcare burden of CMA with comprehensive cost‐effectiveness modeling, and to investigate the impact of clinical phenotypes and factors such as the timing of diagnosis and approaches to management, which may impact the broader clinical outcomes of children with CMA.

## CONFLICT OF INTERESTS

R. M. and K. E. G. have previously received honoraria from Nutricia, Nestle Health Science, Mead Johnson and Abbott. D.A.‐M. is an honorary Associate Professor at the Institute of Health Informatics, University College London, UK, and an employee of Cegedim Rx, who was funded by Nutricia. to undertake the research. K. S. is employed by Nutricia. A. L. C. and R. J. S., both of whom hold honorary research posts with the University of Southampton, are also employed part‐time by Nutricia.

## ETHICS STATEMENT

Ethical approval for this study was granted by the Scientific Review Committee which reviews all research involving the THIN database (protocol reference number: 20‐009).

## AUTHOR CONTRIBUTIONS

*Conceptualization*: Katy Sorensen, Abbie L. Cawood, and Rebecca J. Stratton. *Methodology*: Katy Sorensen, Abbie L. Cawood, and Dionisio Acosta‐Mena. *Formal analysis*: Dionisio Acosta‐Mena. *Data curation*: Katy Sorensen, Abbie L. Cawood, and Dionisio Acosta‐Mena. *Writing—original draft preparation*: Katy Sorensen. *Writing—review and editing*: Katy Sorensen, Abbie L. Cawood, Rosan Meyer, Kate E. Grimshaw, Dionisio Acosta‐Mena, and Rebecca J. Stratton. All authors have read and agreed to the published version of the manuscript.

## Data Availability

The data that support the findings of this study are available from the corresponding author upon reasonable request.
